# Predictive factors for severe toxicity of sunitinib in unselected patients with advanced renal cell cancer

**DOI:** 10.1038/sj.bjc.6605303

**Published:** 2009-09-15

**Authors:** H Rezaei Kalantari

**Affiliations:** 1 Department of Oncology, C.H. Peltzer - La Tourelle, Verviers, Belgium

**Sir,**

In agreement with Van der Veldt *et al* (2008) and sunitinib clinical trials (Motzer *et al*, 2007), we observe in our clinical practice a significant proportion of patients who ultimately need a sunitinib dose reduction as a consequence of intolerable adverse events that cannot be effectively managed otherwise. Hitherto often unreported, however, is the potential of a dose re-escalation in patients who earlier required a sunitinib dose reduction as a consequence of intolerable adverse events. Such a dose re-escalation is clinically very relevant as a meta-analysis of sunitinib studies previously showed that higher sunitinib plasma levels were associated with a higher probability to achieve an objective response, a longer time to progression and with an improved overall survival (Houk *et al*, 2007). Two recent patient cases from our own practice illustrate the relevance of a dose re-escalation with sunitinib to achieve maximal patient benefit and treatment outcome with this potent yet sometimes challenging drug.

A 71-year-old patient with an imatinib-refractory gastrointestinal stromal tumor started sunitinib therapy at the recommended 50 mg dose (4/2 schedule). After the first treatment cycle, fatigue (grade: 2–3, grading according to the National Cancer Institute Common Terminology Criteria for Adverse Events v3.0), lipothymia (grade 2), anaemia (grade 1), leukopenia (grade 2) and thrombocytopenia (grade 1) were observed. On the basis of these adverse events, the second treatment cycle was tapered off to alternating 37.5 and 25 mg sunitinib daily doses (4/2 schedule). His toxicities disappeared and his Eastern Cooperative Oncology Group (ECOG) performance status (PS) improved from PS 2 to PS 0 during the third cycle at this dosing regimen. Gradual dose re-escalations to 37.5 mg, alternating 50 and 37.5 mg and 50 mg sunitinib daily doses (all 4/2 schedule) were subsequently introduced in the fourth, fifth and sixth treatment cycles, respectively, without any significant toxicity issues. At present, the patient still remains on the 50 mg dose, with good tolerance and disease stabilisation.

A 47-year-old patient with renal cell carcinoma (RCC) was started on sunitinib therapy at the 50 mg once daily (4/2 schedule) regimen. Fatigue, nausea, pruritus, articular erythroderma, epistaxis (all grade 1) and hypertension and pruritus (grade 2) developed, but these were effectively clinically managed and did not require a modification of the sunitinib dose. During the second treatment cycle, therapy appeared to be better tolerated, with observations of mild fatigue, skin cracks and elevated transaminases (all grade 1) as remaining tolerance issues. After the third treatment cycle, a partial response (RECIST criteria) was observed, but tolerability deteriorated with increasing fatigue and skin toxicity (although both are still considered grade 1), and the ECOG PS deteriorated from PS 1 to PS 2. On the basis of these observations, the fourth treatment cycle was down-titrated to alternating 37.5 and 25 mg sunitinib daily doses (4/2 schedule), with improved patient tolerability: no toxicities were reported and the PS improved from 2 to 0. The fifth treatment cycle was continued at this dose, but the patient required hospitalisation for abdominal pain and his PS deteriorated to 2. At that time, the patient asked to cease sunitinib treatment, but we were able to motivate the patient to continue therapy at 25 mg sunitinib daily doses (4/2 schedule). The sixth treatment cycle was associated with a good tolerability and the PS improved to 1, but the CT scans showed that the abdominal pain was associated with renewed tumor growth. The sunitinib dose was therefore re-escalated to 50 mg once daily (4/2 schedule) for the seventh treatment cycle, during which the patient experienced a strong reduction in abdominal pain (no further need for analgesics), a good tolerability and PS 1. In an attempt to further optimise the anti-tumor activity in this young patient, the sunitinib dose was subsequently further up-titrated to 62.5 mg once daily (4/2 schedule) for the eighth treatment cycle, during which the patient experienced no toxicity, an improvement in PS from 1 to 0 and was able to return back to work. This schedule is currently being continued in the tenth treatment cycle with good tolerability, PS 0 and further disease stabilisation.

Although dose reductions as a consequence of intolerable and unmanageable adverse events are not infrequently required in patients on sunitinib, our cases illustrate that it is equally important to consider the potential of dose re-escalations in at least some patients to maximise patient benefit and treatment outcome. Kahl *et al* (2008) and Schöffski *et al* (2009) recently reported similar observations of clinical benefit after sunitinib dose re-escalation or escalation in progressing RCC patients. Hence, in future studies of targeted agents, it will be useful to not only report the frequency of dose reductions but also to report any dose re-escalations in the patient population under study.
